# A Frequent *PNPLA3* Variant Is a Sex Specific Disease Modifier in PSC Patients with Bile Duct Stenosis

**DOI:** 10.1371/journal.pone.0058734

**Published:** 2013-03-07

**Authors:** Kilian Friedrich, Christian Rupp, Johannes Roksund Hov, Niels Steinebrunner, Karl-Heinz Weiss, Adolf Stiehl, Maik Brune, Petra Kloeters Yvonne Schaefer, Peter Schemmer, Peter Sauer, Peter Schirmacher, Heiko Runz, Tom Hemming Karlsen, Wolfgang Stremmel, Daniel Nils Gotthardt

**Affiliations:** 1 Department of Internal Medicine IV, University Hospital of Heidelberg, Heidelberg, Baden-Wuerttemberg, Germany; 2 Department of Internal Medicine I, University Hospital of Heidelberg, Heidelberg, Baden-Wuerttemberg, Germany; 3 Department of General Surgery, University Hospital of Heidelberg, Heidelberg, Baden-Wuerttemberg, Germany; 4 Institute of Pathology, University Hospital of Heidelberg, Heidelberg, Baden-Wuerttemberg, Germany; 5 Institute of Human Genetics, University Hospital of Heidelberg, Heidelberg, Baden-Wuerttemberg, Germany; 6 Norwegian PSC Research Center, Research Institute for Internal Medicine and Department of Gastroenterology, Division for Cancer, Surgery and Transplantation, Oslo University Hospital, Oslo, Norway; 7 Institute of Clinical Medicine, Faculty of Medicine, University of Oslo, Oslo, Norway; 8 Institute of Medicine, Faculty of Medicine and Dentistry, University of Bergen, Bergen, Norway; University College London, United Kingdom

## Abstract

**Background & Aims:**

Primary sclerosing cholangitis predominantly affects males and is an important indication for liver transplantation. The rs738409 variant (I148M) of the *PNPLA3* gene is associated with alcoholic and non-alcoholic liver disease and we evaluated its impact on the disease course of PSC.

**Methods:**

The I148M polymorphism was genotyped in 121 German PSC patients of a long-term prospective cohort and 347 Norwegian PSC patients.

**Results:**

In the prospective German cohort, actuarial survival free of liver transplantation was significantly reduced for I148M carriers (p = 0.011) compared to wildtype patients. This effect was restricted to patients with severe disease, as defined by development of dominant stenosis (DS) requiring endoscopic intervention. DS patients showed markedly decreased survival (p = 0.004) when carrying the I148M variant (I148M: mean 13.8 years; 95% confidence interval: 11.6–16.0 vs. wildtype: mean 18.6 years; 95% confidence interval: 16.3–20.9) while there was no impact on survival in patients without a DS (p = 0.87). In line with previous observations of sex specific effects of the I148M polymorphism, the effect on survival was further restricted to male patients (mean survival 11.9 years; 95% confidence interval: 10.0–14.0 in I148M carriers vs. 18.8 years; 95% confidence interval: 16.2–21.5 in wildtype; p<0.001) while female patients were unaffected by the polymorphism (p = 0.65). These sex specific findings were validated in the Norwegian cohort (p = 0.013).

**Conclusions:**

In male PSC patients with severe disease with bile duct stenosis requiring intervention, the common I148M variant of the *PNPLA3* gene is a risk factor for reduced survival.

## Introduction

Primary sclerosing cholangitis (PSC) is a chronic inflammatory disorder that can progress to liver cirrhosis and liver failure [1], [2]. PSC predominantly affects young males that are at high risk to develop end stage liver disease and has a reported prevalence of up to 16.2 per 100.000 with increasing incidence rates reported [3]. However, disease progression is highly variable [4], [5] and can be complicated by the formation of dominant stenosis (DS). The formation of DS is associated with reduced transplant-free survival [6]. Although the etiology and pathogenesis of PSC are still unknown, increased prevalence among first-degree relatives pointed towards a genetic component [7]. In addition, genome-wide association studies have genetically linked PSC to human leukocyte antigen (HLA) complex as well as non-HLA loci as MST1 (macrophage stimulating 1 gene) and BCL2-like 11 protein [8], [9].

*PNPLA3* is a member of the patatin-like phospholipase family [10] and promotes cellular lipid synthesis by converting lysophosphatidic acid into phosphatidic acid [11]. Its expression is upregulated during feeding and consequently downregulated in the fasted state [12]. The isoleucine to methionine switch at position 148 results in higher lysophosphatidic acid acyltransferase activity. Therefore, the I148M variant causes a gain-of-function effect leading to increased TG synthesis and deposition [11]. GWAS reported a strong association between increased hepatic triglyceride content and the rs738409 variant (I148M) in the *PNPLA3* gene [13]. Nonalcoholic steatohepatitis (NASH) patients with the I148M variant are susceptible to a more aggressive form of the disease and are at greater risk of developing liver fibrosis [14], [15]. A negative correlation exists between the male proportion in the studied populations and the effect of the I148M variant on liver fat content, suggesting that a sexual dimorphism of the I148M variant affects NAFLD development [14]. This observation was further sustained by the strong genetic association of the I148M variant and NAFLD that was reported in women ≤50 years old, but not in women >50 years [16]. The impact of the I148M variant upon NAFLD seems to be best explained by the additive genetic model as shown by a meta-analysis comparing heterozygosity (GC) and wildtype genotype (CC). Evaluation of the risk associated with heterozygosity for the I148M variant and histological severity of NAFLD showed that the effect does not differ when carrying only one G allele compared to the GG genotype. In addition, carriers of only one G allele showed significantly elevated ALT levels when compared with the wildtype genotype (CC) [14].

In this study, we evaluated the impact of the I148M polymorphism on the course of PSC in a prospective study cohort and in a second, independent cohort of Norwegian PSC patients.

## Methods

### Study design

The prospective study began on May 1, 1987, the outcome of all patients in the study was followed up until September 2011. From 121 patients DNA samples were obtained after written consent, so altogether 121 patients were included in this final evaluation of the prospective study cohort (Table 1). The study was approved by the ethics commitee of the University of Heidelberg. Informed consent to participate in the study was obtained from each subject in confirmation with the Ethics Commitee of the University of Heidelberg. The study was carried out in accordance with the Declaration of Helsinki.

**Table 1:** Association between allele frequency (WT = CC, I148M = GC/GG) of the *PNPLA3* rs738409 polymorphism and laboratory/clinical parameters in PSC patients at baseline.

Mean ± SEM	Prospective cohort	Norwegian cohort
N	121	347
Female/Male	38/83	95/252
Age at diagnosis [years]	33.6±12.3	36.4±13.3
IBD	84	279
ERC at institute	121	224
Dominant stenosis	81	123*
Liver transplantation	22	143
Death	7	57
CCA	4	34

* 224 patients of the Norwegian cohort received ERC at the tertiary care centre. Of these 224 patients 123 patients needed endoscopic intervention due to bile duct stenosis.

As described previously [17], selection criteria for enrolling patients with PSC in the prospective study included typical endoscopic retrograde cholangiographic findings, serum alkaline phosphatase activity of at least twice the normal range, negative antimitochondrial antibody titers and a liver biopsy compatible with the diagnosis of PSC. Exclusion criteria were decompensated liver cirrhosis, if liver transplantation was foreseen or diagnosis of cholangiocellular carcinoma occurred within three months after presentation, patients with a history of neoplastic disease and/or hepatic comorbidity.

At entry to the prospective study cohort, physical examination, biochemical blood tests, and abdominal ultrasound was performed, and repeated in yearly intervals. All patients were treated with UDCA. The biliary system was evaluated using endoscopic retrograde cholangiography (ERC) at entry of this study in all the patients. In case DS were present, ERC was repeated yearly. If there was no narrowing of the common duct at entry to this study, ERC was performed every two years up to 1995. Since all the patient with a DS showed increased liver enzymes, starting from 1995 repeat ERC in patients without DS was only performed when alkaline phosphatase or gamma-glutamyltransferase increased by 20% or greater. A DS was defined as a stenosis with a diameter of less than 1.5 mm of the common duct or less than 1.0 mm of a hepatic duct (within 2 cm of the bifurcation). In patients with total or subtotal stenosis of the major duct and biochemical evidence of cholestasis, rigid dilation followed by balloon dilatation of the stenosis was performed after a small endoscopic sphincterotomy of the papilla. In patients with dominant stenosis of the common bile duct, ERC was repeated at yearly intervals.

### Pathology reports of liver explants

Of the patients that had received liver transplantation during the course of the ongoing study, histology was evaluated by two board certified pathologists with special expertise in liver pathology.

### Norwegian study cohort

The second, Norwegian cohort consists of 347 PSC patients with follow-up data available, all recruited at admission to Oslo University Hospital Rikshospitalet, a tertiary care center. Median age of diagnosis was 36.4 years, while the life-time prevalence of inflammatory bowel disease and cholangocarcinoma were 80.4% and 9.7%, respectively. Random healthy controls (n = 1068, 51% male) were selected from the Norwegian Bone Marrow Donor Registry. Information on death for each patient was provided due register. Of the 347 PSC patients, 224 patients received ERC at the tertiary care center (Table 1). All ERCs performed from 1999 on were analyzed in regard of interventions (dilatation/stenting). These interventions were commonly performed due to a DS.

### RT Quantitative PCR for *PNPLA3* expression analysis

Patient tissues and mouse tissue samples were analyzed. Sample preparation was performed in accordance with the local ethics committee. Total RNA from tissues, as well as from the human cell lines Caco, MZCHA1, and HepG2 were isolated using RNeasy kit (Qiagen, Hilden, Germany). cDNA was synthesized from total RNA with oligo(dT) primers by using the OmniscriptcDNA Kit (Qiagen, Hilden, Germany) according to the manufacturer's instructions. Specific mRNA transcripts were quantified using QuantiTect SYBR Green (Qiagen) and the following QuantiTect primers (Qiagen) for murine *PNPLA3* (QT00129710) and for human *PNPLA3* (QT00055419). The murine housekeeping gene Gapdh (QT01658692) and human housekeeping gene Gapdh (QT01192646) were used as the reference. Data acquisition and analysis of gene expression was performed on the LightCycler software package (Light Cycler 480 software release 1.5.0, Roche Mannheim, Germany). Each PCR reaction was run in triplicates. Target mRNA expression was compared to the expression of the housekeeping gene.

### Animal work

Male C57BL/6 mice (Charles River Laboratories, Sulzfeld, Germany) were used at 10 weeks of age. Tissues were harvested and immediately snap frozen in liquid nitrogen and stored at −80°C. All experiments were approved by the Animal Care and Use Committee of the University of Heidelberg.

### *PNPLA3* analysis

For analysis of the I148M *PNPLA3* polymorphism (rs738409), genomic DNA was extracted from whole blood samples applying the QIAamp DNA Blood Midi Kit (Qiagen, Hilden, Germany; #51183) according to the manufacturer's instructions. DNA was then analyzed using the *PNPLA3* kit from TibMolBiol (Berlin, Germany; #11241101) on a Light Cycler 2.0 (Light Cycler software release 3.5.28, Roche, Mannheim, Germany). Briefly, approximately 100 ng of genomic DNA were applied to real time PCR with 95°C for 10 minutes and 45 cycles of temperatures 95, 60 and 72°C for 10, 10 and 15 seconds, respectively. The SNPs were subsequently detected by melting curve analysis, monitoring the fluorescence intensities of SNP-specific DNA probes starting at 35°C. Single signals at either 46 or 57°C revealed the homozygous genotype “G” (“C” on the resulting mRNA) or “C” (“G” on the resulting mRNA), respectively, while signals at both 46 and 57°C were characteristic for heterozygous genotypes.

Genotyping in the Norwegian cohort was performed with both the SequenomMassARRAY® iPLEX® Gold system [18] at the Centre for Integrative Genetics (Norwegian University of Life Sciences, Ås, Norway) and a TaqMan™ SNP Genotyping Assay (Applied Biosystems, Foster City, CA, USA) [19]. In overlapping samples the concordance was 99.6% between these two methods.

### Statistics

Continuous data were compared with the nonparametric Wilcoxon rank-sum test, probability distributions by using Chi-square test. The actuarial survival free of liver transplantation rate was estimated by the Kaplan-Meier estimator. Differences between the actuarial estimates were analyzed using the log-rank test.

## Results

### Prospective study cohort

In the prospective cohort of 121 patients 63.6% (n = 77) were wildtype (CC), 33.1% (n = 40) heterozygous (GC) and 3.3% (n = 4) homozygous for the mutation (GG, Table 2). These data were in Hardy-Weinberg-equilibrium (X^2^ = 0.19). Since the I148M variant of the *PNPLA3* gene follows the additive genetic model, patients carrying either one or two mutated allele were combined for further analyses and compared to patients carrying two wild-type alleles (I148M  =  GC/GG, WT  =  CC). Mayo risk score showed no significant difference based on genotype (p = 0.610).

**Table 2:** Allele frequencies in PSC patients of the prospective study cohort and the Norwegian cohort as well as a Norwegian control population for the rs738409 polymorphism investigated.

*PNPLA3*	Norwegian healthy controls (n = 1068)	Prospective PSC cohort (n = 121)	p*	Norwegian PSC cohort (n = 347)	p*
rs738409 C > G	C = 1687 (79.4%)	C = 194 (80,1%)	0.49*	C = 543 (78.2%)	0.51*
	G = 437 (20.6%)	G = 48 (19,8%)		G = 151 (21.8%)	
	C/C = 676 (63.7%)	C/C = 77 (63,6%)		C/C = 215 (62.0%)	
	C/G = 335 (31.5%)	C/G = 40 (33,1%)		C/G = 113 (32.6%)	
	G/G = 51 (4.8%)	G/G = 4 (3,3%)		G/G = 19 (5.5%)	

* Pearson's chi-square test for differences in allele frequencies.

Genotypes were in Hardy-Weinberg equilibrium (p_prospective_ = 0.19, p_norway_ = 0.43, p_controls_ = 0.26).

The main study endpoint was actuarial survival free of liver transplantation, defining death and liver transplantation as events. 16.9% (n = 13/77) of the WT patients and 36.4% (n  =  16/44) of I148M carriers died or needed liver transplantation. Kaplan–Meier analysis revealed that actuarial survival free of liver transplantation was significantly reduced (p = 0.011) in the presence of the I148M variant (mean 13.9 years; 95% confidence interval: 11.8–16.0) compared to the WT patients (mean 19.3 years; 95% confidence interval: 17.3–21.4; Fig. S1).

### Impact of I148M variant upon dominant bile duct stenosis

**Figure 1:**
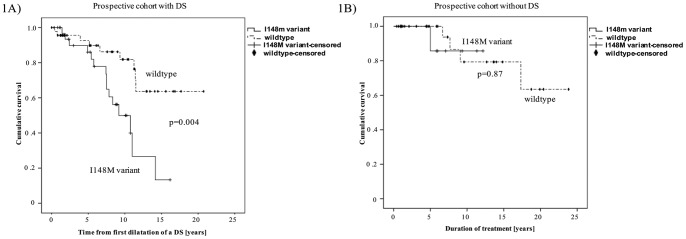
Kaplan – Meier estimate for the I148M variant and dominant stenosis. Figure 1A: In patients with a DS, the I148M variant markedly impairs actuarial survival free of liver transplantation: Kaplan – Meier estimate of all patients in the prospective study cohort with a DS (n = 81) shows markedly impaired actuarial survival free of liver transplantation in the presence of a DS (p = 0.004). Figure 1B: In patients without a DS, the I148M variant does not affect actuarial survival free of liver transplantation: Kaplan–Meier estimate of all patients in the prospective study cohort without a DS (n = 40) showed no difference in actuarial survival free of liver transplantation in the presence of a DS (p = 0.87).

There was a trend towards development of DS for I148M patients (p = 0.167) without reaching statistical significance. However, Kaplan-Meier analysis showed that patients with a DS showed significantly impaired actuarial survival free of liver transplantation (p = 0.004) when carrying either one or two mutated alleles (mean 9.6 years; 95% confidence interval: 7.7–11.5) compared to WT patients (mean 16.2 years; 95% confidence interval: 13.7–18.6; Fig. 1A). Interestingly, patients without a DS were not affected by the I148M variant (mean 11.1 years; 95% confidence interval: 9.2–13.0) compared to WT patients (mean 19.5 years; 95% confidence interval: 15.9–23.0) in regard of actuarial survival free of liver transplantation (p = 0.87; Fig. 1B). Since predominantly patients with a DS were affected by the I148M polymorphism, we included patients with a DS for further analysis of gender specific differences.

### Sexual dimorphism of the I148M variant

**Figure 2:**
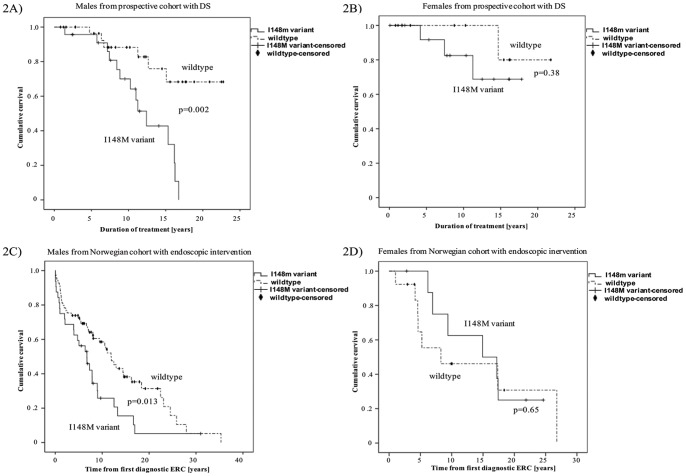
Gender specific Kaplan–Meier estimate for the I148M variant. Figure 2A: In male PSC patients with a DS, the I148M variant impairs actuarial survival free of liver transplantation in the German prospective study cohort: Kaplan-Meier estimate of all male PSC patients with a DS (n = 55) in the prospective study cohort shows markedly impaired actuarial survival free of liver transplantation (p = 0.002) when carrying the I148M genotype (n = 23) compared to male wildtype patients (n = 32). Figure 2B: In female PSC patients with a DS, the I148M variant does not affect actuarial survival free of liver transplantation in the German prospective study cohort: Kaplan-Meier estimate of all female PSC patients with a DS (n = 26) in the prospective study cohort shows no difference in actuarial survival free of liver transplantation (p = 0.38) when carrying the I148M genotype (n = 10) compared to the wildtype genotype (n = 16). Figure 2C: In male PSC patients with endoscopic intervention, the I148M variant impairs actuarial survival free of liver transplantation in the Norwegian study cohort: Kaplan-Meier estimate of all male PSC patients with endoscopic intervention (n = 101) in the Norwegian study cohort shows markedly impaired actuarial survival free of liver transplantation (p = 0.013) when carrying the I148M genotype (n = 32) compared to the wildtype genotype (n = 69). Figure 2D: In female PSC patients without endoscopic intervention, the I148M variant does not affect actuarial survival free of liver transplantation in the Norwegian study cohort: Kaplan-Meier estimate of all female PSC patients with endoscopic intervention (n = 22) in the Norwegian study cohort shows no difference in actuarial survival free of liver transplantation (p = 0.65) when carrying the I148M genotype (n = 9) compared to the wildtype genotype (n = 13).

Kaplan-Meier Analysis revealed a sex specific effect of the I148M variant upon actuarial survival free of liver transplantation. Male I148M patients with a DS showed significantly impaired (p = 0.002; Fig. 2A) actuarial survival (mean 11.9 years; 95% confidence interval: 10.0–13.9) compared to male WT patients (mean 18.8 years; 95% confidence interval: 16.2–21.5). In contrary, there was no impact of the I148M variant in female patients (Female_I148M carriers: mean 20.3 years; 95% confidence interval: 17.8–22.8 vs. Female_WT: mean 14.8 years; 95% confidence interval: 11.9–17.7; Fig. 2B).

### Norwegian study cohort

We screened for the I148M polymorphism in a second, independent cohort of 347 Norwegian PSC patients. The overall callrate in the Norwegian cohort was 99.5%. The allele frequencies were similar between PSC patients and healthy controls (Table 2). There were 62.0% (n = 215) wildtype patients, 32.6% (n = 113) were heterozygous and 5.5% (n = 19) homozygous for the mutation (Table 2). The genotype frequencies were in Hardy-Weinberg equilibrium. 224 of the 347 PSC patients received ERC at the tertiary care center in Norway. 123 of these PSC patients received endoscopic intervention (dilatation/stenting) due to stenosis of the biliary system.

In this second, independent cohort of PSC patients, we also detected a gender specific impact of the I148M polymorphism on actuarial survival free of liver transplantation. While Male_I148M carriers showed significantly impaired actuarial survival (mean 7.8 years; 95% confidence interval: 4.9–10.6) compared to Male_WT patients (mean 13.4 years; 95% confidence interval: 10.5–16.3; p = 0.013; Fig. 2C), actuarial survival in female PSC patients did not differ when carrying the I148M variant (mean 15.1 years; 95% confidence interval: 10.4–19.8) compared to wildtype patients (mean 13.4 years; 95% confidence interval: 6.9–19.9; Fig. 2D).

**Figure 3:**
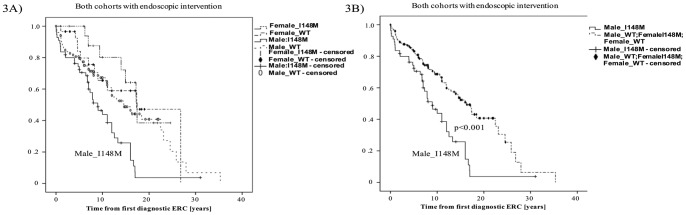
Combined gender specific Kaplan–Meier estimate for both cohorts. Figure 3A: Gender specific combined analysis of the prospective cohort and the Norwegian cohort: Kaplan-Meier estimate of male and female PSC patients of the German prospective cohort and Norwegian study cohort with endoscopic intervention (n = 204). Male I148M patients with a DS (n = 55) of both cohorts show markedly impaired actuarial survival free of liver transplantation when carrying the I148M genotype (p<0.001) compared to Male_WT (n = 101), Female_I148M (n = 19), Female_WT (n = 29) patients of both cohorts. Figure 3B: Gender specific combined analysis of the prospective cohort and the Norwegian cohort: Kaplan-Meier estimate of male I148M patients with endoscopic intervention compared to combined analysis of Male_WT, Female_I148M, Female_WT patients of both cohorts with endoscopic intervention (n = 204). Kaplan-Meier estimate: male PSC patients with a DS (n = 55) shows markedly impaired actuarial survival free of liver transplantation when carrying the I148M genotype (p<0.001) compared to the other patients with endoscopic intervention of both study cohorts (n = 149).

Combined analysis of PSC patients of the prospective study cohort and the Norwegian study cohort that had received endoscopic intervention due to biliary stenosis (n = 204) demonstrates the gender specific impact of the I148M variant on PSC patients. Male_I148M patients show significantly impaired actuarial survival free of liver transplantation (p<0.001) compared to Male_WT patients while the polymorphism does not affect female PSC patients (Fig. 3A–B).

### Expression analysis

Using RT-PCR we were able to show that the *PNPLA3* gene is expressed in the biliary system in humans, mice and in cholangiocyte cell culture lines (Fig. S2A–B).

## Discussion

Primary sclerosing cholangitis is a cholestatic liver disease that predominantly affects young males and is caused by chronic inflammation of the bile ducts that can progress to liver cirrhosis, liver failure or cholangiocarcinoma [1]. Its hallmark is the chronic inflammation that can lead to the obstruction of the bile ducts. Therapeutic approaches, including endoscopic dilatation and treatment with ursodesoxycholic acid might delay disease progression, but the only potential cure is liver transplantation [1], [3]. In addition, PSC patients are at risk for developing hepatobiliary carcinoma with a cumulative lifetime incidence of 10–15% [20]. The etiology of PSC however remains unknown. As previously reported in detail, there is an association between the *PNPLA3* I148M variant with alcoholic or non-alcoholic steatohepatitis [21]. The variant has also been associated with higher triglyceride levels in hepatocytes, an effect that seems to follow a gender specific pattern [14]. However, there is no information on a contribution to cholestatic liver diseases.

The I148M variant represents a susceptibility factor for hepatic fibrosis in patients with chronic liver diseases [21], but has only been attributed to parenchymal liver disease. In the present study, there was no evidence for marked steatosis in liver explants of transplanted patients carrying the mutated allele. *PNPLA3* is known to be expressed in various human tissues, with highest expression levels in the liver [22]. In line with the detection of *PNPLA3* gene expression in the biliary system of humans, mice and in a human cholangiocyte cell culture line (Fig. S2A–B), we next sought to assess whether the I148M variant affects disease course of PSC patients. Therefore, we evaluated the clinical impact of the I148M variant in a well-characterized prospective study cohort of 121 PSC patients with a maximum follow-up of up to 25 years and in a second, independent Norwegian cohort of 347 PSC patients with particular attention to gender specific differences.

Using Kaplan–Meier analysis, we demonstrated that in the German prospective cohort carriers of the I148M *PNPLA3* variant show significantly reduced actuarial survival free of liver transplantation (p = 0.011) compared to wildtype patients. The hallmark of PSC is periductal fibrosis that can lead to the formation of DS, a feature of disease progression and associated with reduced survival resulting in priorisation of PSC patients with DS on waiting list for liver transplantation. Since the presence of a DS is associated with reduced transplant-free survival and has been implemented in liver allocation [6], we evaluated whether the impact of the I148M polymorphism on actuarial survival is attributed to the formation of DS. Although we observed a trend for development of dominant bile duct stenosis (p = 0.167) in I148M patients, this did not reach statistical significance. However, patients diagnosed with a DS showed markedly reduced actuarial survival free of liver transplantation (p = 0.004) when carrying the I148M variant (mean 9.6 years; 95% CI: 7.7–11.5; Fig. 1B) from the time-point, when a DS was first diagnosed and treated compared to wildtype patients (mean 16.2 years; 95% CI: 13.7–18.6). Interestingly, the I148M variant did not affect actuarial survival in patients without a DS (p = 0.87; Fig. 1C) suggesting that the I148M *PNPLA3* variant particularly affects PSC patients with severe bile duct strictures.

Since a sexual dimorphism of the I148M variant upon NAFLD development has been reported [14] and the clinical finding of PSC is predominant in males, we investigated gender specific differences of the I148M polymorphism in PSC patients with a DS. While actuarial survival of male PSC patients was significantly reduced for carriers of the I148M variant (p = 0.002; Fig. 2A), female patients were not affected by the I148M mutation (p = 0.38; Fig. 2B). This goes in line with a recent Meta-analysis evaluating the impact of the I148M variant upon NAFLD stating a negative correlation between the male proportion in the studied populations and the effect of the I148M variant on liver fat content [14].

In order to reassess these gender specific findings, we screened a second, independent cohort of 347 Norwegian PSC patients for the I148M variant. In this cohort, 234 PSC patients received ERC at the tertiary institute of which 123 PSC patients needed endoscopic intervention due to bile duct stenosis. In congruence to the findings of the prospective study cohort, male carriers of the I148M variant showed significantly reduced actuarial survival free of liver transplantation (p = 0.013; Fig. 2C) compared to male wildtype patients. Again, the I148M polymorphism did not affect female PSC patients with bile duct stenosis (p = 0.651; Fig. 2D).

By screening 469 PSC patients for the I148M *PNPLA3* variant, we were able to detect a gender specific impact of the I148M polymorphism on PSC patients, particular in the presence of dominant bile duct stenosis. These results sustain and affiliate studies that previously reported a sexual dimorphism for both PSC and the I148M variant of the *PNPLA3* gene. Based on the findings of our study, male carriers of the I148M variant show significantly reduced actuarial survival free of liver transplantation in the presence of a DS. These results were validated in a second, independent cohort of PSC patient. Therefore, male carriers of the I148M variant can be identified as a high risk PSC subgroup, which is pivotal for proper risk stratification, surveillance strategies and liver transplant allocation.

Up to now *PNPLA3* function has been largely restricted to hepatocytes and adipocytes. The presented data of the sex specific impact of the *PNPLA3* variant upon PSC patients might be the basis for further studies. According to our results, genetic testing for the common *PNPLA3* variant could improve diagnostic and therapeutic approaches in patients with primary sclerosing cholangitis, particular in the presence of a DS.

## Supporting Information

Figure S1 **Kaplan – Meier estimate for PSC patients regarding the I148M variant.** The I148M variant is associated with reduced actuarial survival free of liver transplantation in PSC patients: Kaplan – Meier analysis estimate of all patients in the prospective study cohort (n = 121). There were 16 events for I148M carriers and 13 events for WT patients showing a significantly reduced actuarial survival free of liver transplantation for carriers of the I148M polymorphism (p = 0.011).
(TIF)

Figure S2 **PNPLA3 mRNA gene expression data.** Figure S2A: PNPLA3 mRNA is expressed in the biliary tissue of mice: PNPLA3 mRNA expression in liver, gallbladder, spleen and heart tissues of C57Bl/6 mice was obtained using RT-PCR. Data represents means ± SD (n = 3 per group). Figure S2B: PNPLA3 mRNA is expressed in human liver and biliary cell lines: PNPLA3 mRNA in HepG2 and MZCHA1 cell lines was obtained using RT-PCR. Three experiments were performed. Assays were performed in triplicates and are representative of three independent experiments. Values are the means ± SD.
(TIF)
