# Supplementary figures and images for: A Frequent PNPLA3 Variant Is a Sex Specific Disease Modifier in PSC Patients with Bile Duct Stenosis
Source: PLoS One. 2013 Mar 7;8(3):e58734. doi: 10.1371/journal.pone.0058734 (PMC3591368; doi:10.1371/journal.pone.0058734)

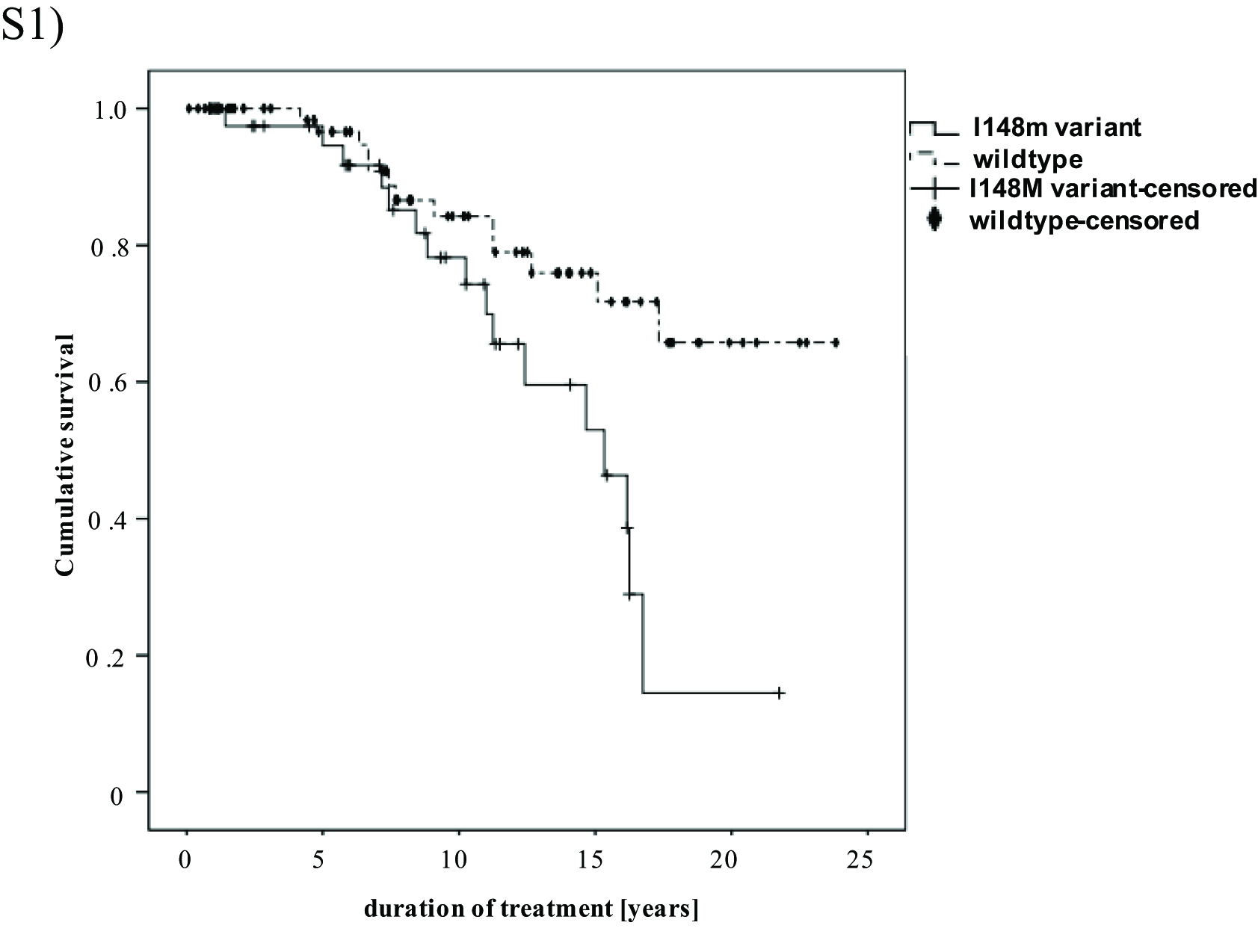

Supplement: Figure S1 — Kaplan – Meier estimate for PSC patients regarding the I148M variant. The I148M variant is associated with reduced actuarial survival free of liver transplantation in PSC patients: Kaplan – Meier analysis estimate of all patients in the prospective study cohort (n = 121). There were 16 events for I148M carriers and 13 events for WT patients showing a significantly reduced actuarial survival free of liver transplantation for carriers of the I148M polymorphism (p = 0.011). (TIF) [file pone.0058734.s001.tif]

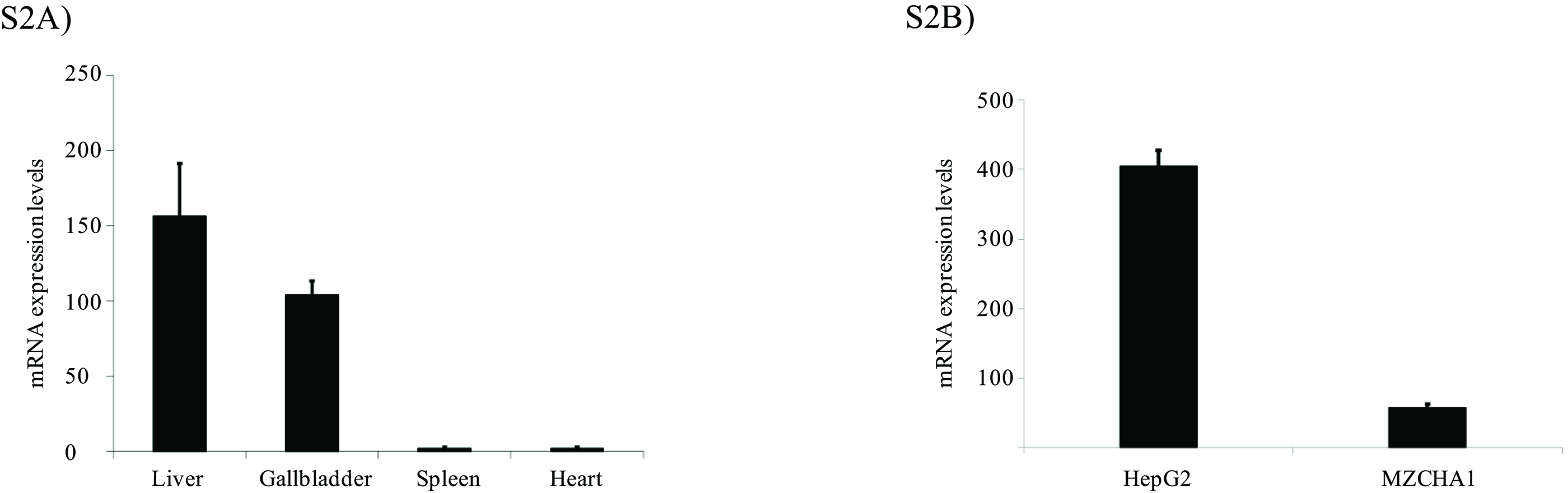

Supplement: Figure S2 — PNPLA3 mRNA gene expression data. Figure S2A: PNPLA3 mRNA is expressed in the biliary tissue of mice: PNPLA3 mRNA expression in liver, gallbladder, spleen and heart tissues of C57Bl/6 mice was obtained using RT-PCR. Data represents means ± SD (n = 3 per group). Figure S2B: PNPLA3 mRNA is expressed in human liver and biliary cell lines: PNPLA3 mRNA in HepG2 and MZCHA1 cell lines was obtained using RT-PCR. Three experiments were performed. Assays were performed in triplicates and are representative of three independent experiments. Values are the means ± SD. (TIF) [file pone.0058734.s002.tif]
